# Supplementary material for: Cellular interplay via cytokine hierarchy causes pathological cardiac hypertrophy in RAF1-mutant Noonan syndrome
Source: Nat Commun. 2017 May 26;8:15518. doi: 10.1038/ncomms15518 (PMC5458545; doi:10.1038/ncomms15518)
Supplement: Supplementary Information — Supplementary Figures and Supplementary Tables. [file ncomms15518-s1.pdf]

Supplementary Table 1. Additional echocardiographic parameters.

Cre Genotype	Mlc2v				Fsp1				Tie2			
	Baseline		2wk TAC		Baseline		2wk TAC		Baseline		2wk TAC	
	Control	L613V	Control	L613V	Control	L613V	Control	L613V	Control	L613V	Control	L613V
n	29	11	20	8	18	7	16	9	24	12	36	11
HR (bpm)	490 ± 10	520 ± 20	548 ± 7	577 ± 8*	507 ± 12	490 ± 22	550 ± 6	577 ± 23	512 ± 12	532 ± 11	556 ± 6	561 ± 10
SV (μL)	42 ± 1	44 ± 2	31 ± 1	34 ± 2	40 ± 1	39 ± 1	31 ± 1	29 ± 2	42 ± 1	58 ± 2*	30 ± 1	37 ± 2*
EF (%)	63 ± 1	73 ± 2*	42 ± 2	47 ± 5	51 ± 2	47 ± 2	43 ± 2	42 ± 4	63 ± 1	58 ± 2*	41 ± 2	40 ± 2
FS (%)	34 ± 1	42 ± 2*	20 ± 1	24 ± 4	26 ± 1	24 ± 1	21 ± 1	21 ± 2	34 ± 1	31 ± 1	20 ± 1	20 ± 1
CO (mL/min)	20 ± 1	22 ± 1	17 ± 1	20 ± 1*	20 ± 1	19 ± 1	17 ± 1	16 ± 1	21 ± 1	31 ± 1*	17 ± 1	21 ± 1*

HR, heart rate; SV, stroke volume; EF, ejection fraction; FS, fractional shortening; CO, cardiac output. All measurements are means ± S.E.M. * $P < 0.05$ vs. respective controls; two-tailed Student's *t* test.

Supplementary Table 2. Additional hemodynamic parameters.

Cre Genotype	Mlc2v				Fsp1				Tie2			
	Baseline		2wk TAC		Baseline		2wk TAC		Baseline		2wk TAC	
	Control	L613V	Control	L613V	Control	L613V	Control	L613V	Control	L613V	Control	L613V
n	39	16	20	8	18	7	18	11	27	13	36	11
HR (bpm)	533 ± 6	550 ± 13	519 ± 10	534 ± 11	484 ± 10	500 ± 15	514 ± 8	500 ± 23	516 ± 9	521 ± 14	522 ± 8	512 ± 17
LVP (mmHg)	124 ± 2	114 ± 3*	173 ± 4	174 ± 7	112 ± 3	110 ± 5	180 ± 4	178 ± 4	123 ± 3	107 ± 3*	173 ± 3	178 ± 4
EDP (mmHg)	5 ± 1	5 ± 1	17 ± 2	17 ± 4	6 ± 1	5 ± 1	19 ± 2	14 ± 2	7 ± 1	6 ± 1	19 ± 2	18 ± 2
SysP (mmHg)	120 ± 3	110 ± 5*	170 ± 3	170 ± 8	108 ± 3	106 ± 5	178 ± 4	175 ± 4	120 ± 3	101 ± 3*	169 ± 3	175 ± 4
DiaP (mmHg)	87 ± 2	80 ± 3*	73 ± 1	71 ± 3	79 ± 2	74 ± 5	73 ± 2	73 ± 3	86 ± 2	74 ± 3*	71 ± 1	72 ± 4
-dP/dt (mmHg/s)	-9950 ± 240	-11030 ± 550*	-8620 ± 360	-8560 ± 590	-9560 ± 280	-9950 ± 390	-9000 ± 430	-9540 ± 510	-9820 ± 160	-9880 ± 310	-9050 ± 410	-9320 ± 330

LVP, left ventricular pressure; EDP, end-diastolic pressure; SysP, systolic pressure; DiaP, diastolic pressure. All measurements are means ± S.E.M. * $P < 0.05$ vs. respective controls; two-tailed Student's *t* test.

Supplementary Table 3. Primers for qRT-PCR

Gene	Primer sequences
Tnf	Forward- 5’ CAGTTCTATGGCCCAGACC 3’
	Reverse- 5’ CTTGGTGGTTTGCTACGAC 3’
Il6	Forward- 5’ TCTATACCACTTCACAAGTCGGA 3'
	Reverse- 5’ GAATTGCCATTGCACAACTCTTT 3’
Atp2a2	Forward- 5’ ATTACAGCCTTTGTAGAGCC 3’
	Reverse- 5’ CATTTCTTTCCTGCCACAC 3’
Gapdh	Forward- 5’ TCCTTTAGGATTTGGCCGT 3’
	Reverse- 5’ TTGATGGCAACAATCTCCAC 3’

Supplementary Figure 1

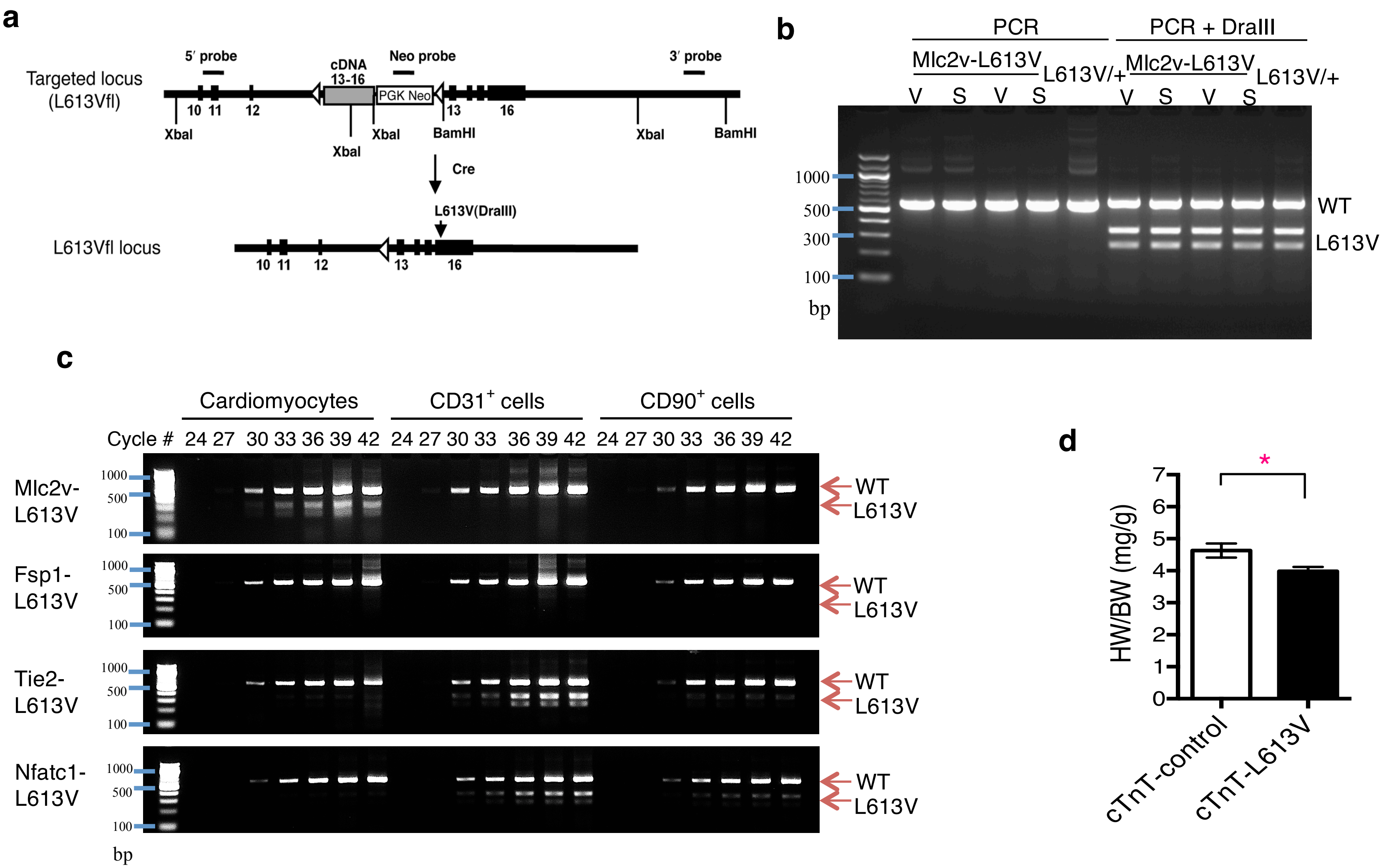

Supplementary Figure 1. Assessment of lineage-specific expression of Raf1 mutant. **a**, Targeting strategy for inducible *Raf1*^{L613V} knock-in mice. Figure adapted from Wu *et al.*[1]. **b**, *Raf1*^{L613V} is expressed at similar levels in hearts of Mlc2v-L613V and global knock-in mice. RNA was isolated from the left ventricle (V) or septum (S) of two Mlc2v-L613V hearts or the total heart of a global knock-in (L613V/+) mouse, and reverse transcribed into cDNA. The PCR product of the resulting cDNA was digested with DraIII, indicating *Raf1*^{L613V} expression. **c**, Similar to **b**. RNA was extracted from isolated CMs, CD31^{+}, CD90^{+} cells to assess specificity of Cre lines. **d**, No increase in HW/BW ratio in *cTnT*Cre: *Raf1*$^{L613Vfl/+}$ mice (mean ± S.E.M.; n = 7 (cTnT-control); 5 (cTnT-L613V); * $P < 0.05$, two-tailed Student's t test).

Supplementary Figure 2.

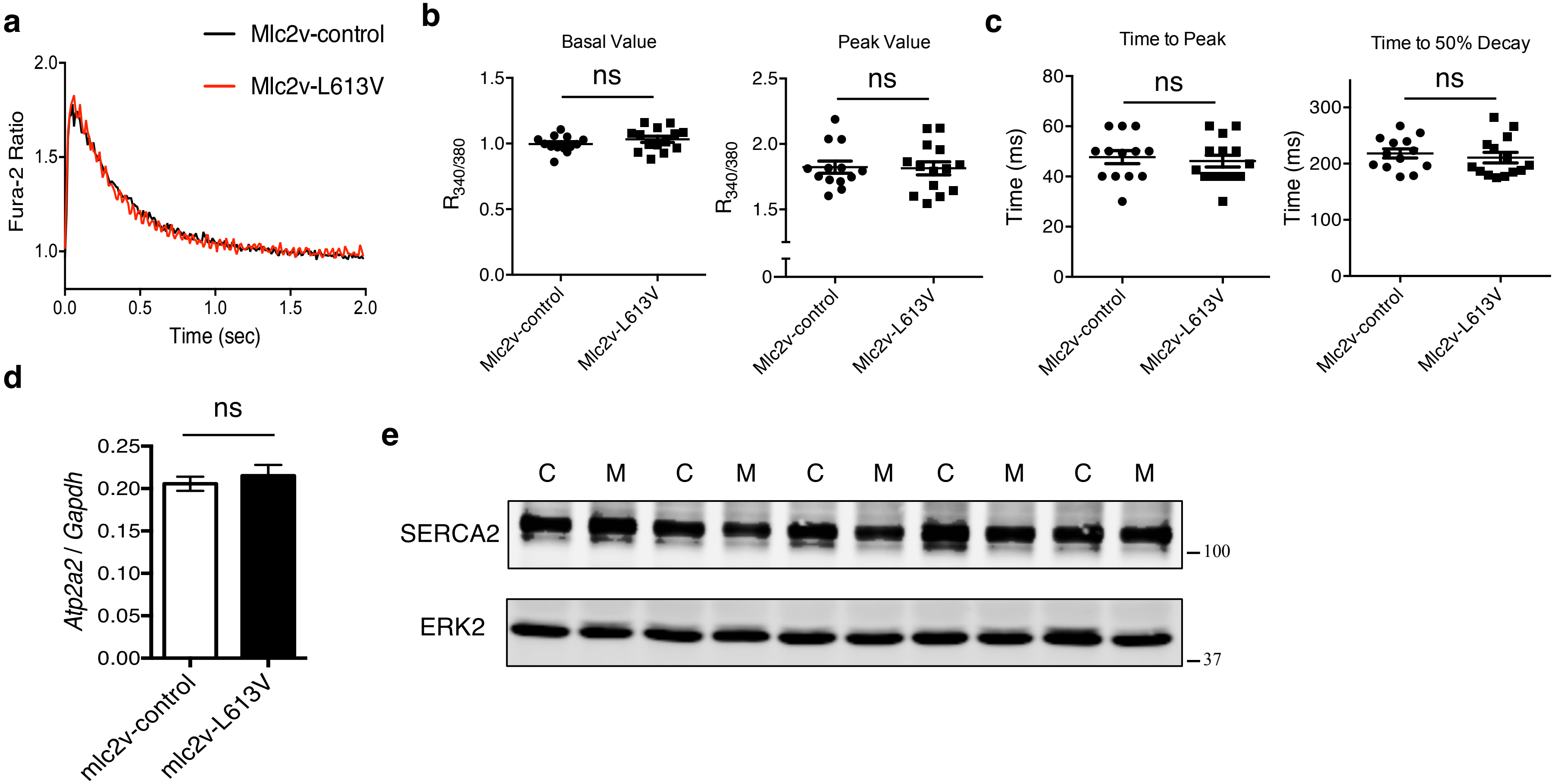

Supplementary Figure 2. No difference in calcium transient in CM-restricted RAF1 mutants. **a-c**, Ca^{2+} transients in freshly isolated single adult ventricular CMs. Fura-2 fluorescence was measured in freshly isolated, single ventricular CMs superfused with Kreb's Hemseleit solution containing 1.2 mM free Ca^{2+}. CMs were field-stimulated at 0.5 Hz, and Ca^{2+} transients were recorded using single cell imaging. **a**, Representative traces of superimposed Ca^{2+} transients in control and Mlc2v-L613V CMs. **b**, Basal and peak values of Ca^{2+} transients, **c**, Time required for peak of Ca^{2+} transient to occur after field stimulation and time to 50% decay of Ca^{2+} transients are shown for the two groups of mice (mean ± S.E.M; n=17 cells in each group from 7 mice in each group; statistical significance was assessed using two-tailed Student's *t* test). **e** and **f**, no difference in *Atp2a2* (encoding SERCA) mRNA, assessed by qRT-PCR (mean ± S.E.M, n=6 (Mlc2v-control) and 7 (Mlc2v-L613V); statistical significance was assessed using two-tailed Student's *t* test). (**e**), or SERCA protein levels, assessed by immunoblotting (**f**), between Mlc2v-control (C) and -L613V mutants (M).

Supplementary Figure 3.

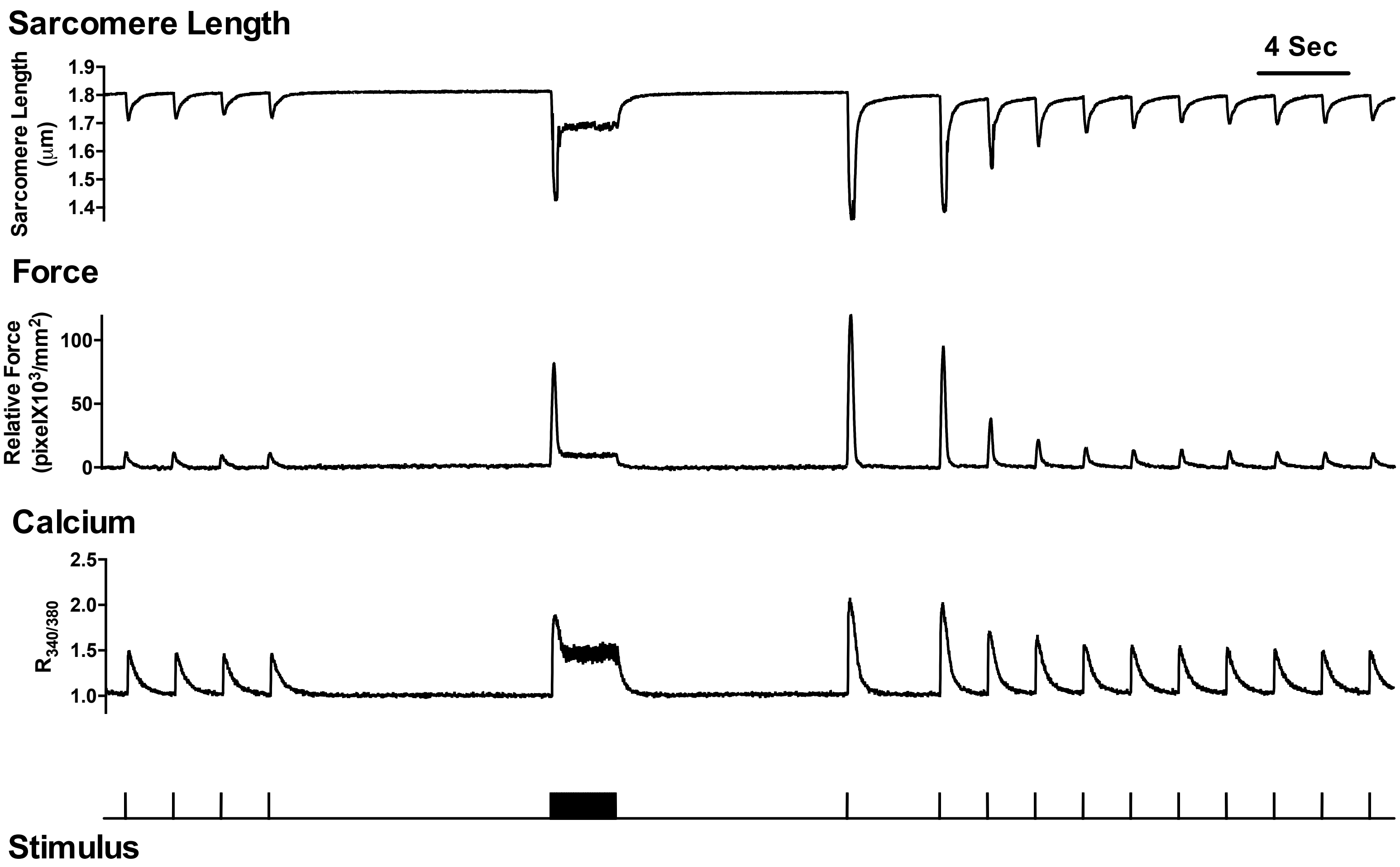

Supplementary Figure 3. Simultaneous force, Ca^{2+} and sarcomere length measurements in single isolated CMs during twitches following field stimulation. Values were recorded as a function of time in isolated CMs attached to glass rods (see Methods). Force levels were varied by rapidly stimulating CMs at 10 Hz for ~2 seconds, followed by a pause for 5 seconds, followed by resumption of field stimulation. Note that the first contraction (corresponding to trace 1 of Supplementary Fig. 4) after the rest period yielded a much larger peak force than that generated before the rapid stimulation ("rest potentiation"). This increase in peak force is associated with a much larger peak Ca^{2+} transient and a much large amount of peak internal sarcomere length shortening. With further repeated stimulation, the peak force (as well as peak Ca^{2+} and peak sarcomere length shortening) decays back to the baseline levels (traces 2-5 of Supplementary Fig. 4), a phenomenon called post-rest potentiation decay. This maneuver allows assessment of the force-Ca^{2+} relationship for twitches under a large range of peak force and peak Ca^{2+} levels.

Supplementary Figure 4.

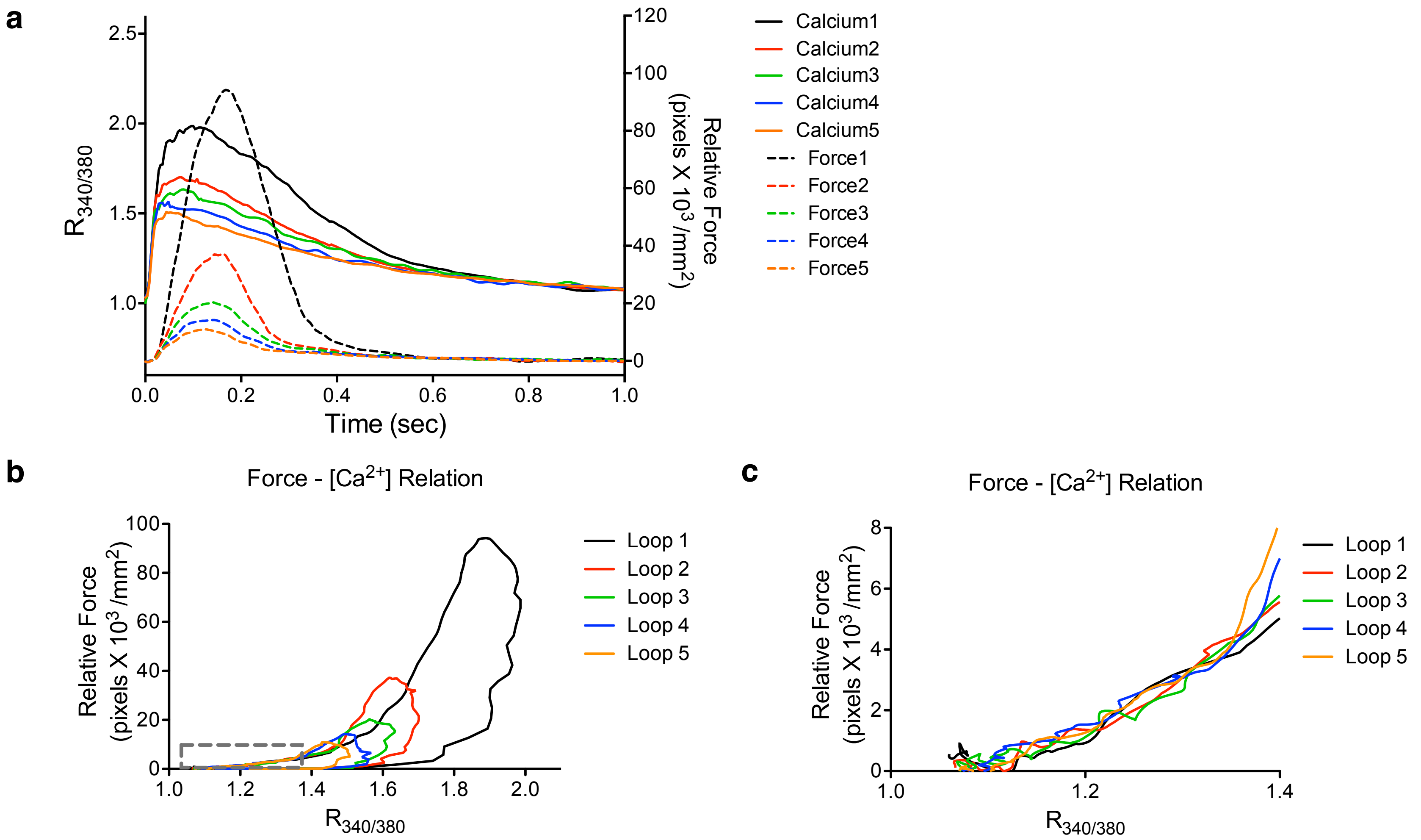

Supplementary Figure 4. Relationship between force and Ca^{2+} during field stimulation. **a**, Superimposed force and Ca^{2+} traces. Peak force and Ca^{2+} levels were generated as described in Supplementary Fig. 3. Traces are color-coded and represent measurements of different twitches generated during post-rest potentiation and decay. **b**, Superimposed force-Ca^{2+} relationships (phase plots) for the traces (1-5) shown in **a**. Note that in the late phase of relaxation, when the sarcomere length has nearly returned to its diastolic resting length of 1.8 µm, phase plots of the force-Ca^{2+} relationship converge onto a single relationship (i.e., the phase plots superimpose exactly, suggesting a steady state is reached independent of the peak force level generated during the twitch). **c**, magnification of the force-Ca^{2+} traces during the late phase of relaxation, illustrating more clearly the converging nature of the force-Ca^{2+} relationship.

Supplementary Figure 5.

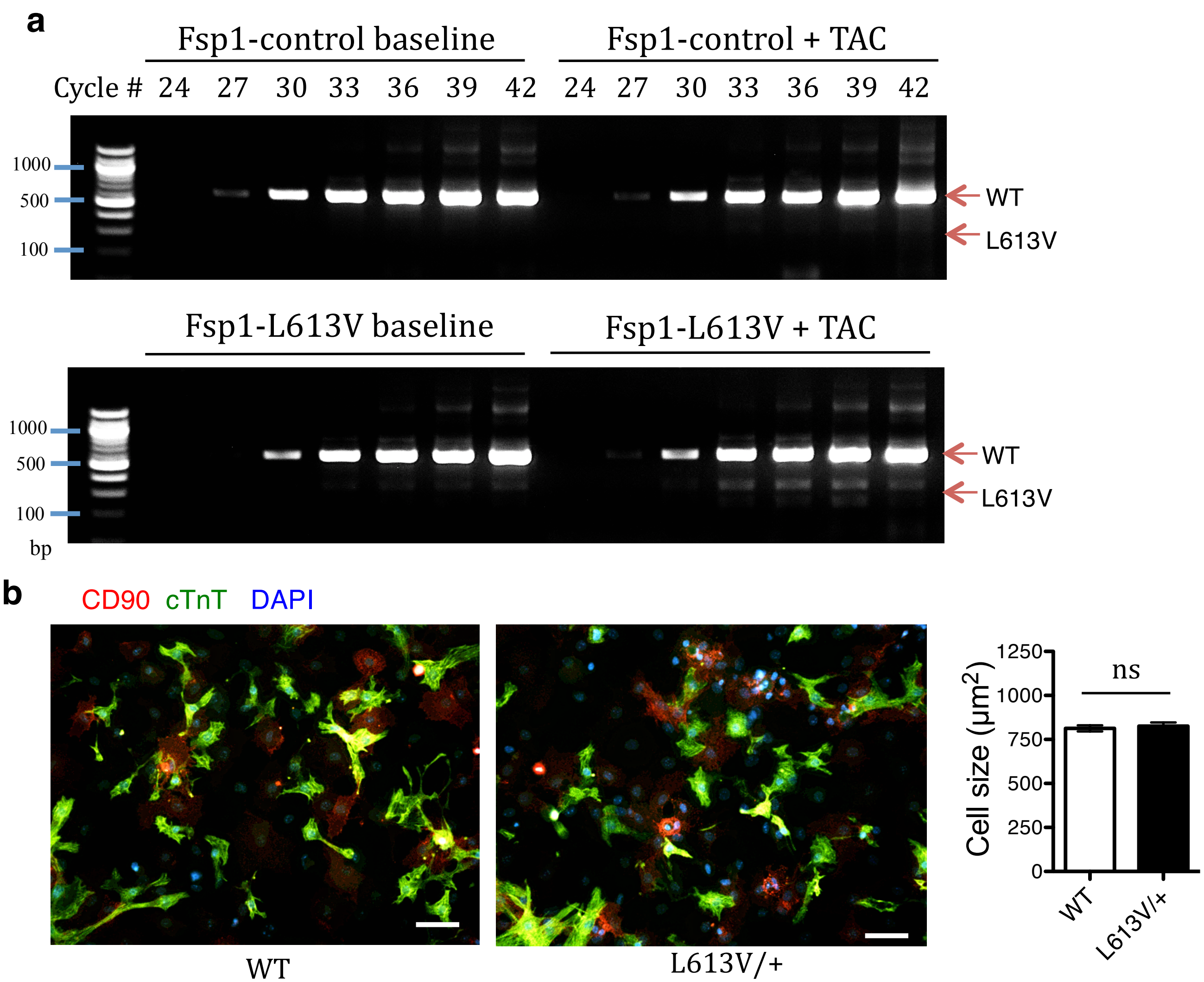

Supplementary Figure 5. Raf1 mutant expression in fibroblasts does not promote CM hypertrophy *in vitro*. **a,** *Raf1^{L613V}* expression is induced in hearts of Fsp1-L613V mice following TAC. RNA was isolated from total heart of Fsp1-control or –L613V mice with or without TAC, and reverse transcribed into cDNA. The PCR product of the resulting cDNA was digested with DraIII, indicating *Raf1^{L613V}* expression. **b**, CD90^{+} cells were isolated from *Raf1^{L613V}* knock-in mice. Left: Representative immunofluorescence staining showing comparable myocyte size following co-culturing with RAF1 control vs. mutant CD90^{+} cells (original magnification, ×200, scale bar: 50 μm). Right: Quantification of one of three independent experiments with similar results.

Supplementary Figure 6.

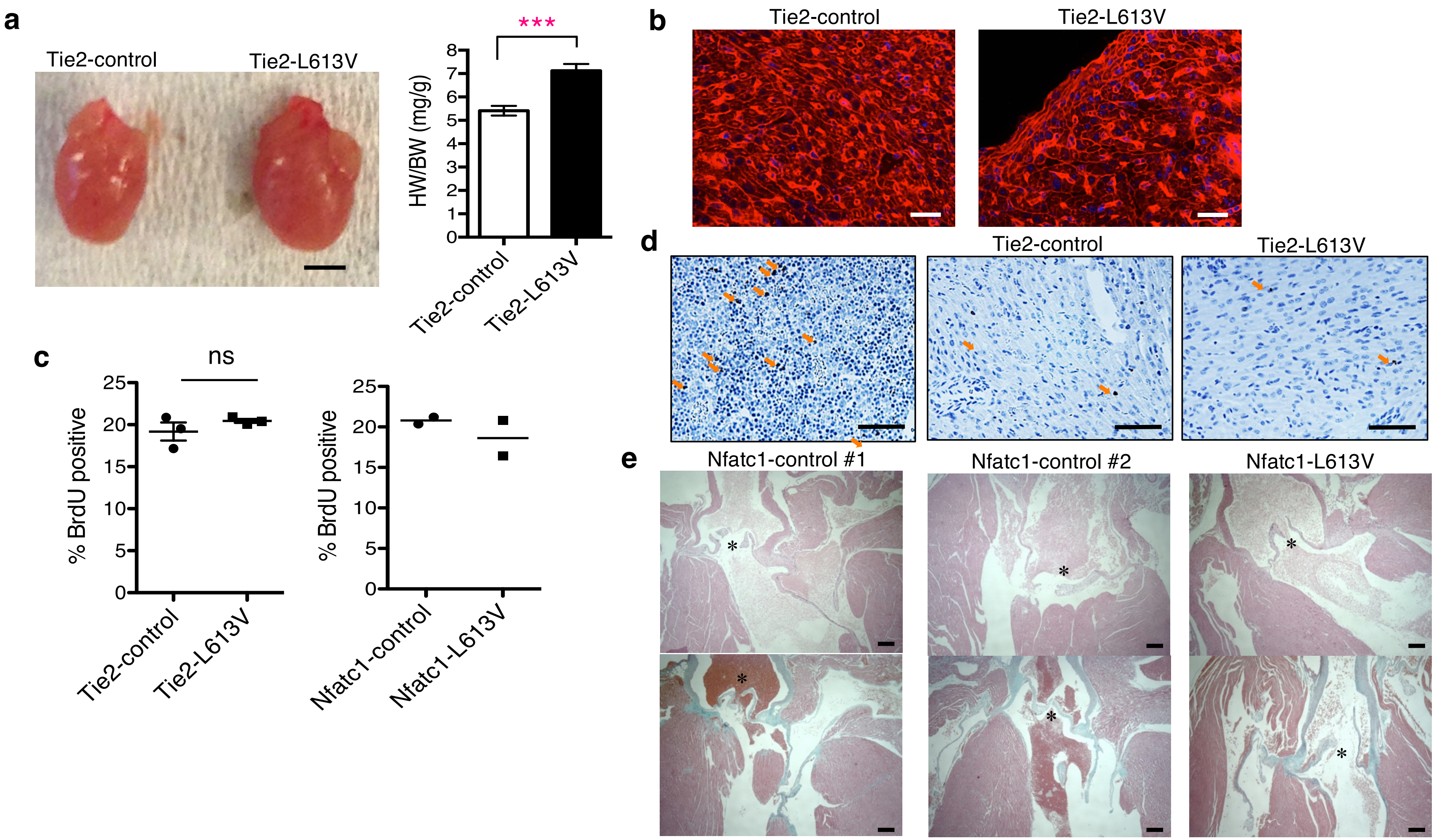

Supplementary Figure 6. a, Representative gross morphology (scale bar: 1 mm) and HW/BW of Tie2-L613V and control hearts at postnatal day 4 (mean ± S.E.M.; n = 16 (Tie2-control) or 7 (Tie2-L613V); $^{***}P < 0.0001$, two-tailed Student's t test). **b**, Representative WGA-stained cross-sections of hearts at postnatal day 4 from Tie2-L613V and control mice (original magnification, ×400, scale bars: 25 μm). **c**, Tie2- and Nfatc1-L613V hearts show similar levels of BrdU incorporation compared to littermate controls at E16.5 (Left: mean ± S.E.M.; n = 3 (Tie2-control) or 3 (Tie2-L613V); two-tailed Student's t test; Right: mean; n = 2 (Nfatc1-control), or 2 (Nfatc1-L613V)). **d**, Tie2-L613V and control hearts show comparable TUNEL staining (original magnification, ×400, scale bars: 50 μm). Liver staining is shown as positive control. Arrows indicate TUNEL^{+} cells. **e**, H&E and Masson's Trichrome staining of serial heart sections, showing normal aortic valves (asterisks) in Nfatc1-L613V mice at week 10 (original magnification, ×50, scale bars: 200 μm).

Supplementary Figure 7.

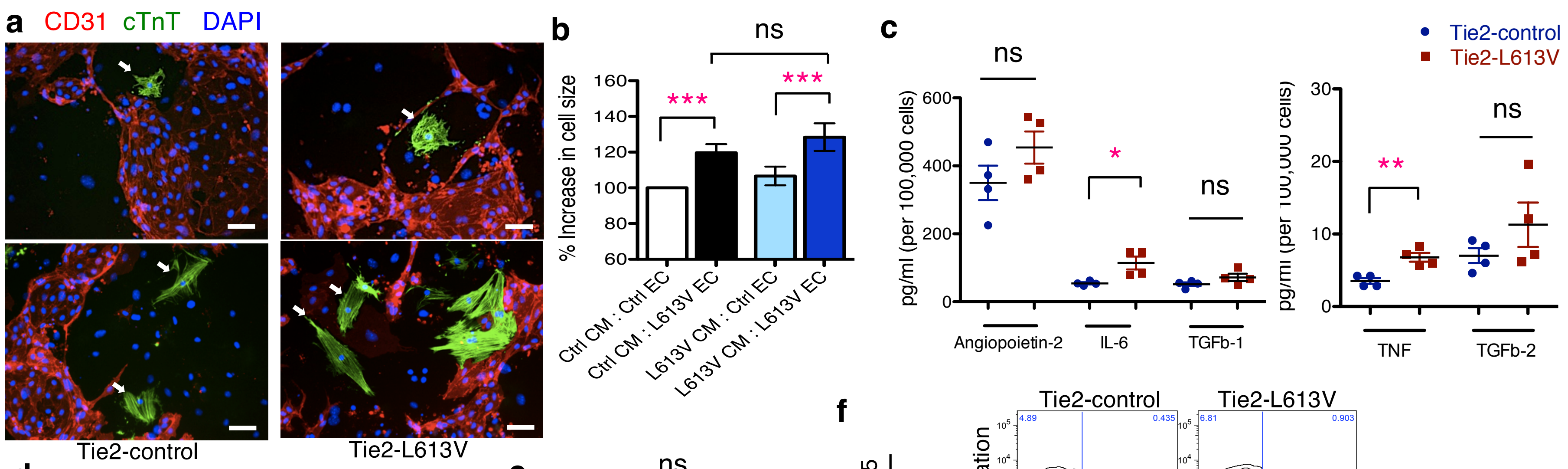

d

	Tie2-control	Tie2-L613V	log2 (L613V/ control)
**Ahsg**	62.3	26.4	**-1.2**
**Sfrp2**	11	4.7	**-1.2**
**Ace2**	0.7	0.3	**-1.2**
**Eln**	431.7	225.6	**-0.9**
**Npr3**	18.8	10	**-0.9**
**Adamtsl2**	42	24.3	**-0.8**
**Angpt1**	3.8	2.3	**-0.7**
**Nppa**	88.4	149	**0.8**
**Timp1**	72.7	134.2	**0.8**
**Cd163**	1.4	3.2	**1.2**
**Il18**	3	7.6	**1.3**
**S100a8**	29	74.2	**1.4**
**Spp1**	25.8	69.3	**1.4**
**Mmp27**	2	5.5	**1.5**
**Mmp12**	6.5	18.4	**1.5**
**Osm**	0.3	0.9	**1.5**
**S100a9**	15.3	46	**1.6**
**Mmp13**	0.4	1.3	**1.6**
**Tnf**	1.4	4.3	**1.6**
**Fcgr2b**	3	9.5	**1.7**
**Mmp8**	3.6	12.8	**1.8**

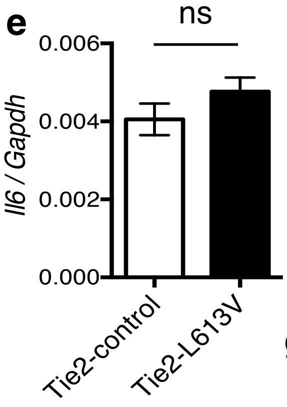

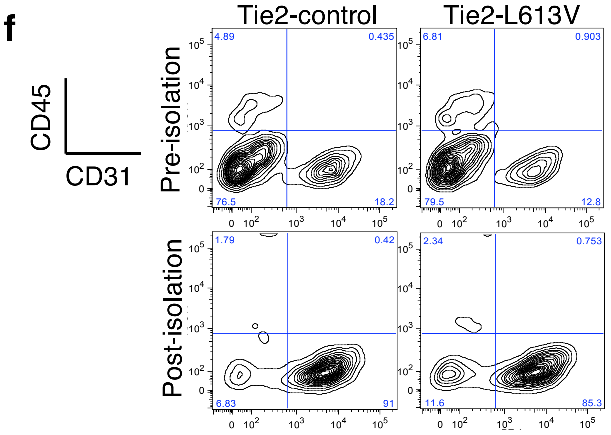

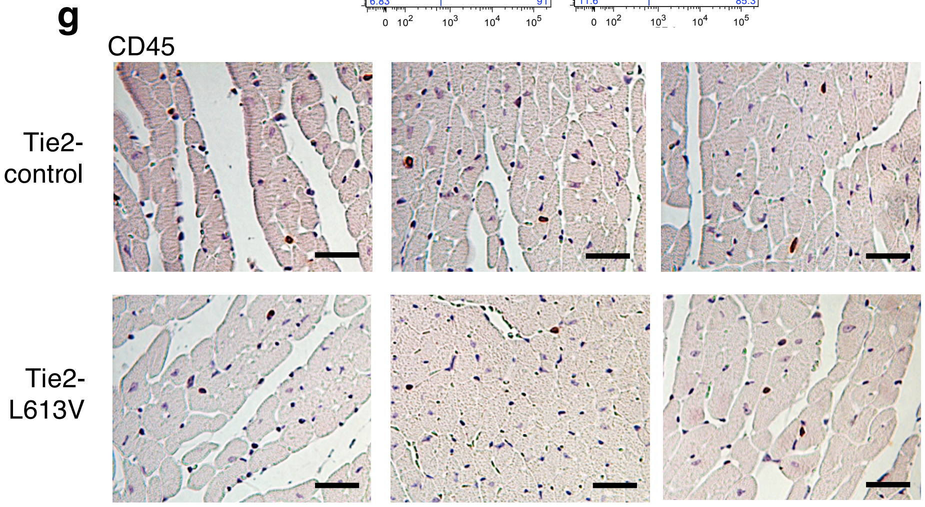

Supplementary Figure 7. a, Additional images showing immunofluorescence staining for CD31 (magenta), cardiac troponin T (green) and DAPI (blue) in co-cultures (original magnification, ×200, scale bars: 50 µm). Arrows indicate individual CMs that were among those assessed for cell size by ImageJ. **b**, RAF1 mutant CMs co-cultured with mutant ECs display no additional hypertrophy. Quantification of one of three independent experiments with similar results (mean ± S.E.M.; 300 CMs counted per group; *** $P < 0.001$, two-tailed Mann-Whitney test). **c**, Cytokine levels in EC-conditioned media, measured by Luminex assays (mean ± S.E.M.; $n = 4$ per group; * $P < 0.05$, ** $P < 0.001$, two-tailed Student's t test). **d**, RNAseq data indicating differentially expressed genes for secreted proteins in ECs ($n = 4$ per group). **e**, No difference in *Il6* transcript levels in mutant and control ECs, assessed by qRT-PCR (mean ± S.E.M.; $n = 7$ per group; statistical significance was assessed using two-tailed Student's t test). **f**, Flow cytometric analysis of primary ECs, showing staining for CD31 and CD45 before and after isolation. Similar results were obtained in an additional experiment. **g**, Tie2-L613V show comparable CD45 staining as their littermate controls (original magnification, ×400, scale bars: 25 µm).

Supplementary Figure 8.

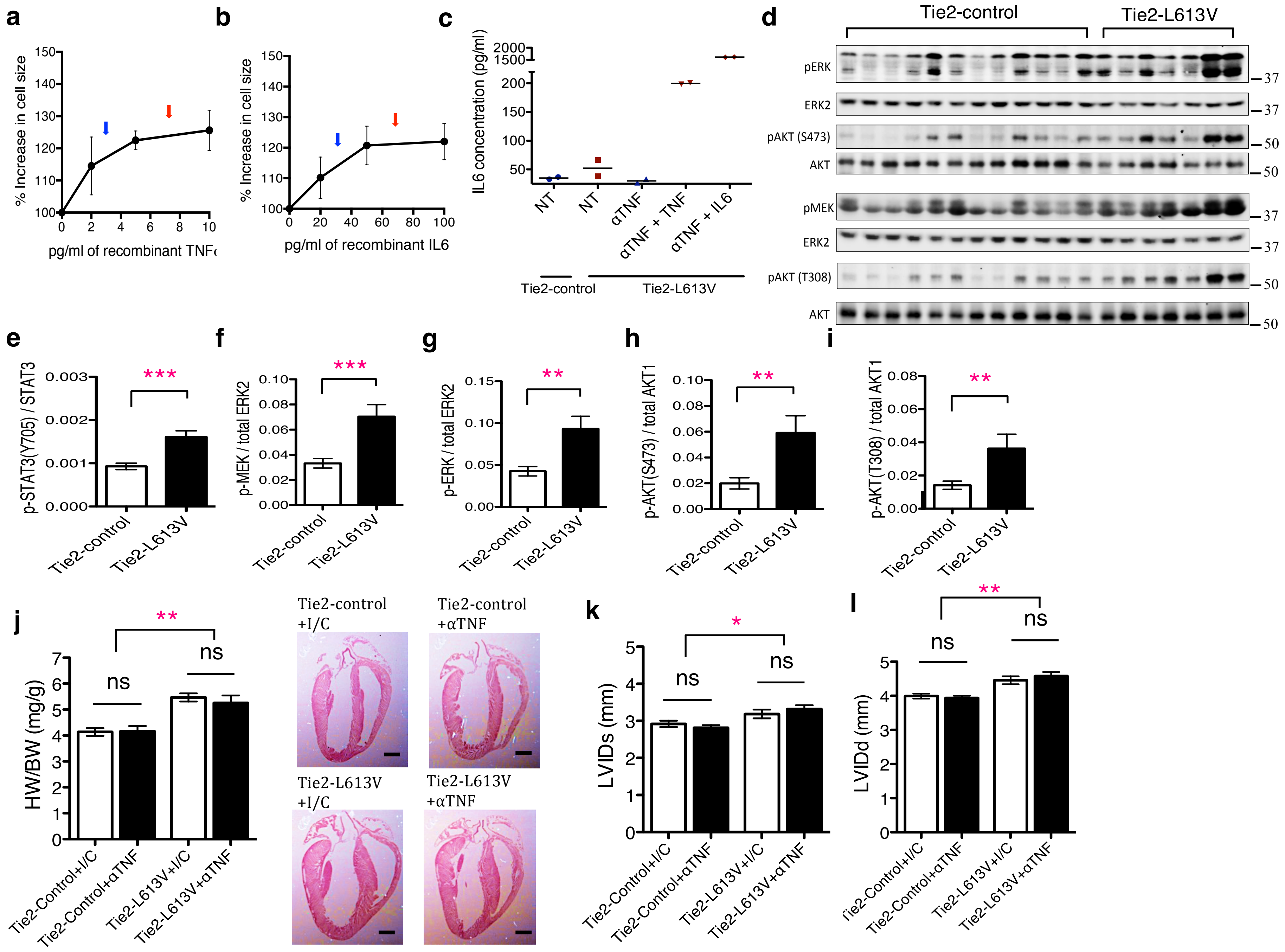

Supplementary Figure 8. **a** and **b**, WT CMs, treated for three days with recombinant TNF (**a**) or IL6 (**b**) at the indicated doses. Data were pooled from two independent experiments, with 200 CMs for each condition quantified using ImageJ. Arrows indicate concentrations detected in control (blue) and mutant (red) EC-conditioned media. **c**, IL6 levels (measured by ELISA) in anti-TNF antibody-treated co-culture media. Note that anti-TNF antibody treatment reduces IL6 production, which increases upon addition of excess TNF. Anti-TNF antibody treatment plus 25 ng/ml IL6 (Ab + IL6) serves as a positive control (mean; n = 2; assays performed in duplicate). **d**, Additional immunoblots from the same experiment as in Fig. 5h. **e**, Quantification of immunoblots shown in Fig. 5h (***P < 0.001, two-tailed Student's t test). **f**-**i**, Quantification of immunoblots in **d** (**P < 0.005; ***P < 0.001, two-tailed Student's t test). **j**, Left: HW/BW after 6 weeks of anti-TNF Ab treatment (mean ± S.E.M.; n = 6 (Tie2-control + isotype control), 5 (Tie2-control + anti-TNF Ab), 4 (Tie2-L613V + isotype control) or 6 (Tie2-L613V + anti-TNF Ab)). Right: Representative H&E staining (scale bar: 1 mm). **k** and **l**, LVIDs and LVIDd, measured by echocardiography at the end of anti-TNF Ab treatment (mean ± S.E.M.; n = 13 (Tie2-control + isotype control), 15 (Tie2-control + anti-TNF Ab), 9 (Tie2-L613V + isotype control) or 12 (Tie2-L613V + anti-TNF Ab); * P < 0.05, **P < 0.005, Bonferroni's post-test when ANOVA was significant).

Supplementary Figure 9.

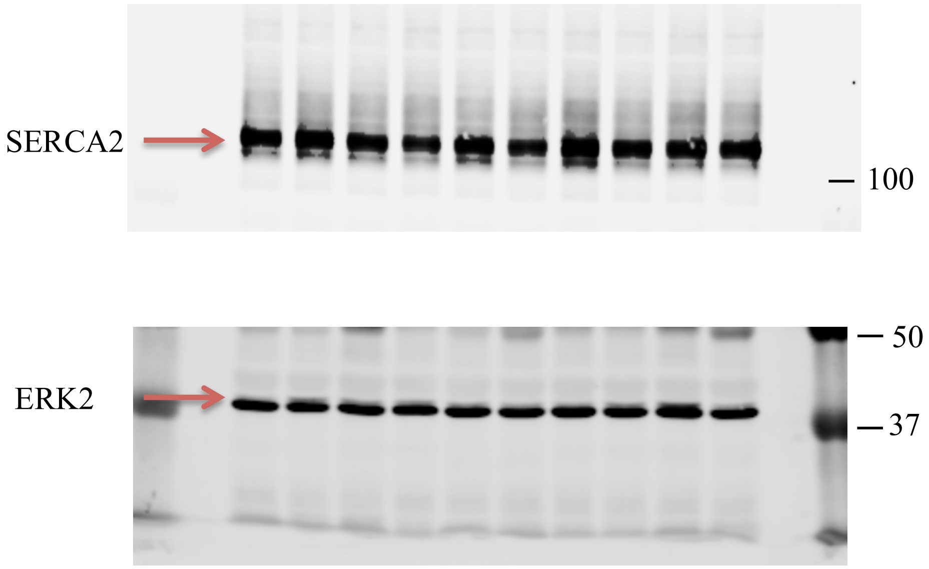

Supplementary Figure 9. Uncropped immunoblots for Supplementary Fig. 2e.

Supplementary Figure 10.

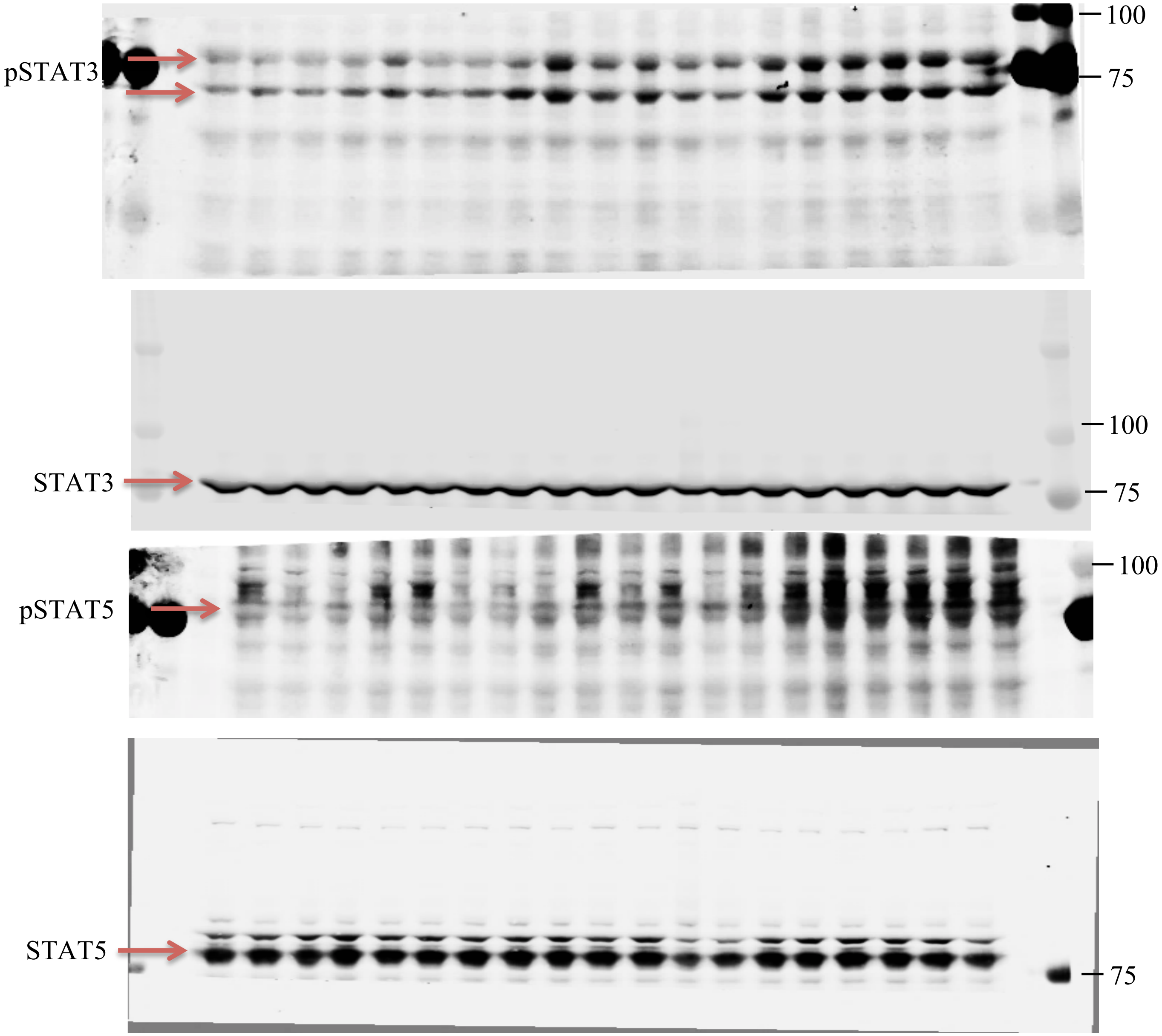

Supplementary Figure 10. Uncropped immunoblots for Fig. 5h.

Supplementary Figure 11.

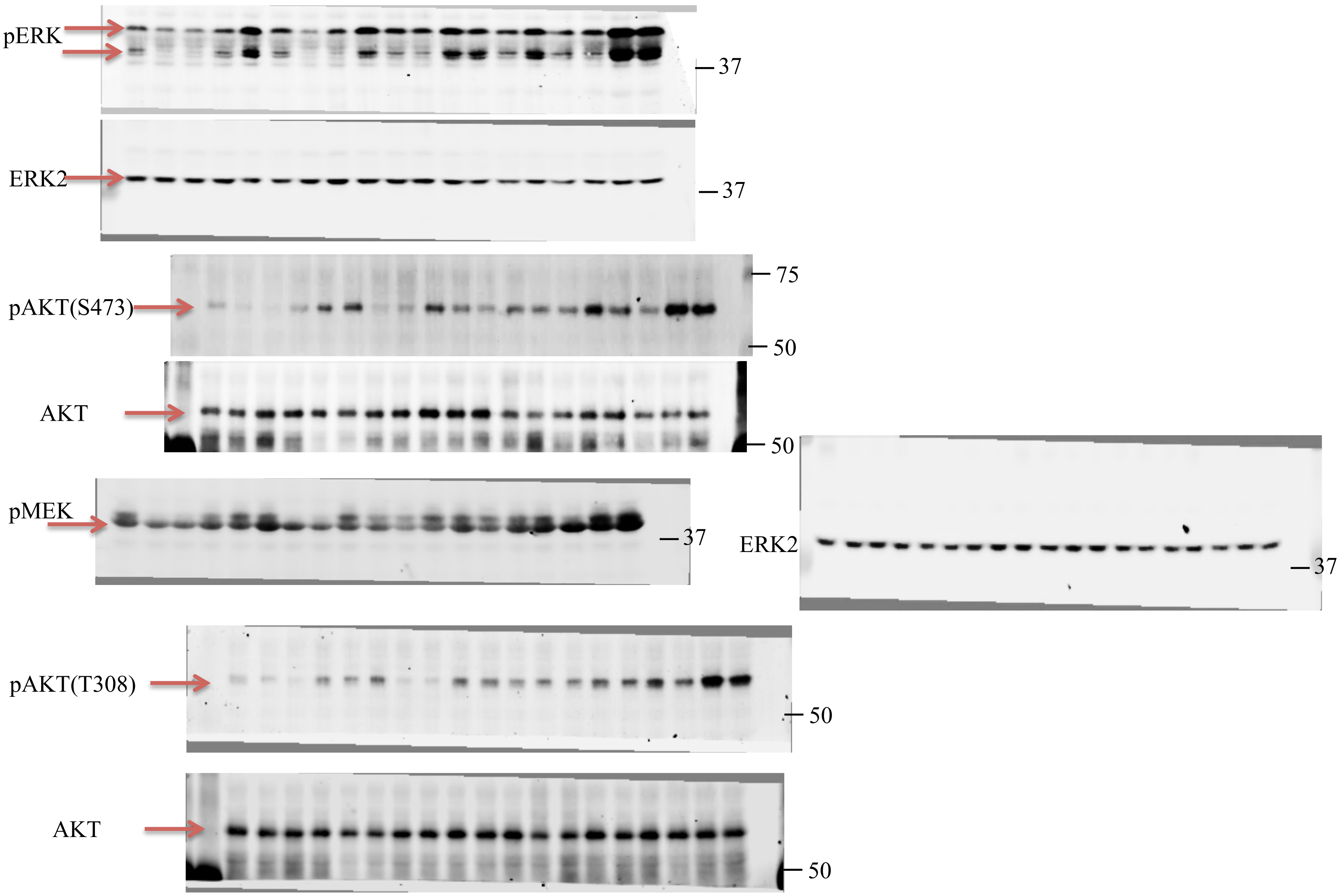

Supplementary Figure 11. Uncropped scans of immunoblots in Supplementary Fig. 8d.
